# Less motor (re-)planning requires fewer working memory resources

**DOI:** 10.1007/s00221-022-06491-8

**Published:** 2022-10-25

**Authors:** Christoph Schütz, Thomas Schack

**Affiliations:** 1 Faculty of Psychology and Sports Science, Bielefeld University, 33615 Bielefeld, Germany; 2 Center for Cognitive Interaction Technology, Bielefeld University, 33619 Bielefeld, Germany; 3 Research Institute for Cognition and Robotics, Bielefeld University, 33615 Bielefeld, Germany

**Keywords:** Motor planning, Motor hysteresis, Working memory, Interference, Recency

## Abstract

In the current study, we asked if less motor re-planning requires fewer resources in working memory (WM). To this end, participants executed a spatial WM task in parallel to different sequential motor tasks: (1) a randomised task with a high amount of motor re-planning and (2) an ordered task with a lower amount of motor re-planning. Recall performance in the spatial WM task was measured as the dependent variable. Hand posture was used to calculate the percentage of motor re-planning and, thus, to validate the experimental manipulation. The percentage of motor re-planning was lower in the ordered task, while spatial WM performance was higher. This indicates that WM resources depleted by the motor task scale with the amount of motor re-planning. Results further showed a significant recency effect (i.e. better recall of late items) in the spatial WM task. As previous studies found that recency effects in a verbal WM task are disrupted by a concurrent motor task, the presence of recency in the current study indicates a differential interference of a concurrent motor task on verbal vs. spatial recall, which has important implications for several current models of WM.

## Introduction

When executing a movement sequence, such as typing a word on a computer keyboard, sequence information has to be retained in working memory (WM) until it is converted into a motor programme. Neurophysiological evidence for this retention was given by single neuron recordings in the dorsal premotor cortex of monkeys, during the execution of a movement sequence (Ohbayashi et al. 2003). The authors found neurons that became active only while their respective movement was executed, and neurons that stayed active until their movement was executed, indicating a temporal storage of sequence information in WM.

One influential model of WM, the *multicomponent model* (Baddeley and Hitch 1974), proposes three distinct WM components: a *central executive*, which divides and shifts attention between WM tasks, and two domain-specific short-term stores, the *phonological loop* and the *visuospatial sketchpad*. Verbal information is stored in the phonological loop, visual and spatial information in the visuospatial sketchpad (Baddeley 2001). The idea of domain-specific stores is supported by a number of studies: execution of hand (Lawrence et al. 2001; Spiegel et al. 2013) and eye movements (Lawrence et al. 2001; 2004) has a larger disruptive effect on spatial than on verbal recall. This selective interference indicates that spatial WM is more closely linked to movement execution (Logie and Pearson 1997). Therefore, a spatial recall task was used to test for disruptive effects of motor planning on WM in the current study.

Several recent reviews that focussed on the interaction of visual WM and action execution emphasised the close bidirectional link between both systems (cf. Heuer et al. 2020; Olivers and Roelfsema 2020; van der Stigchel and Hollingworth 2018). The authors consider visual WM an integral part of the eye (and hand) movement system: attended sensory representations in visual WM are linked to the motor system; their neural activity is transiently enhanced by recurrent feedback. Thus, attention moderates neural plasticity and modulates sensory-action links. Several studies confirmed that the execution (Hanning and Deubel 2018; Heuer and Schubö 2018; Ohl et al. 2017; Ohl and Rolfs 2020) or just the planning (Hanning et al. 2015) of goal-directed eye or hand movements modulates the contents of visual WM. In the inverse direction, the contents of visual WM have been found to affect motor execution (Bahle et al. 2018).

Due to this bidirectional link, a task with a greater amount of motor planning should deplete more WM resources and, thus, have a larger disruptive effect on spatial recall. To test this, in the current study, we had participants perform a sequential motor task (open a column of drawers) in parallel to a spatial WM task (memorise symbols in a 4 × 4 matrix). One feature of sequential motor tasks is that the amount of motor planning can be varied experimentally, due to a behaviour termed *motor hysteresis* (Kelso et al. 1994): In ordered, repetitive movement sequences, participants persist in their former postures. For example, in descending sequences of drawers, participants adopt a pronated posture at the top drawer and persist in a more pronated posture for all subsequent drawers (as compared to ascending sequences; Schütz et al. 2011; Schütz and Schack 2013).

This persistence in the previous posture indicates a partial reuse of the previous motor plan (Rosenbaum et al. 1992) and, thus, a lower amount of motor planning. Based on the size of the hysteresis effect, one can compute the percentage of (motor plan) reuse (PoR; Schütz et al. 2016; Schütz and Schack 2013), as a larger hysteresis effect equals a larger PoR. In a previous study, the PoR in a sequential, ordered drawer task was found to be 15% (Schütz and Schack 2019). This means 85% of each motor plan was created by novel planning. In contrast, in a sequential, randomised task, hysteresis effects were absent (Schütz et al. 2011; Schütz and Schack 2019), which shows that each motor plan was created 100% from scratch.

In a randomised motor planning task, we, therefore, expected a larger depletion of WM resources than in an ordered task (due to a smaller PoR) and, consequently, a larger disruptive effect on recall in the spatial WM task. As a validity check for the conditions, hand pro/supination was measured as a second dependent variable and used to calculate the PoR. We expected a larger PoR in the ordered condition. We further asked whether an announcement of each drawer number affected recall performance or motor planning. In all previous studies, drawer numbers were announced individually in the randomised conditions, whereas participants completed the ordered conditions without announcements (Schütz et al. 2011; Weigelt et al. 2009).

The announcement could draw attentional resources (Cowan 2001; Kahneman 1973) or create another serial episodic record (Jones et al. 1995) or perceptual-motor object (Macken et al. 2015) and, thus, interfere with memory (decreasing recall performance) or motor planning (increasing PoR). Alternatively, the announcement of the upcoming drawer could render retention of the last position in the sequence unnecessary, which could simplify retention in the memory task (increasing recall performance). Finally, the delay between movements caused by the announcement could result in a decay (Jax and Rosenbaum 2007, 2009) of the former motor plan (decreasing PoR). To isolate the effect of the announcement in the current study, we tested randomised and ordered sequences of trials with and without announcement, respectively.

As a second, minor research question, we asked whether recency effects would be present in the current study. Our memory task comprised a sequence of symbols in a spatial matrix. In the literature, bowed serial position curves are well documented for verbal (Brown et al. 2007; Glanzer and Cunitz 1966; Murdock Jr 1962) and spatial (Farrand et al. 2001; Farrand and Jones 1996; Jones et al. 1995) sequences of items: recall is best at the beginning (*primacy effect*) and end (*recency effect*) of a sequence. Three previous studies found that, when a verbal recall task was combined with a concurrent motor task, the recency effect was lost (Logan and Fischman 2011, 2015; Weigelt et al. 2009). This is quite interesting, as it indicates an interference of the concurrent task specifically with the memory processes associated with recency.

In a recent study, Schütz and Schack (2020) combined either a verbal or a spatial memory task with the same motor task and were able to reproduce this loss of recency in the verbal task. In the spatial task, in contrast, the authors found a clear recency effect. This finding suggests that spatial WM tasks, which are more susceptible to interference by a concurrent motor task than verbal WM tasks (Lawrence et al. 2001; 2004; Spiegel et al. 2013), are less susceptible in their recency effect. If this result was reproducible, it would have important implications for at least two current WM models: In the *multicomponent model* (Baddeley and Hitch 1974), recency is commonly linked to the *episodic buffer* (Baddeley 2000), a limited-capacity store of the *central executive* that, due to its *non-domain-specific encoding*, can integrate information from both domain-specific stores (Baddeley 2003). Thus, there should be no differential interference of the same motor task on verbal and spatial recency.

A second WM model, the *object-oriented episodic record* (Jones 1993), has abolished the dedicated-systems view of Baddeley in favour of a unitary, perceptual-motor view, based on findings that verbal and spatial serial recall shows similar position curves and susceptibility to interference (Farrand et al. 2001; Farrand and Jones 1996; Jones et al. 1995). The model assumes a common representation of verbal and spatial information, that is created by perceptual input and motor output processes (Jones et al. 2004; Macken et al. 2015). Interference of a secondary task is a function of the degree to which both tasks contain serial order cues. The model, therefore, cannot account for a differential interference of the same serial task on verbal and spatial recency.

In contrast to the loss of recency in a verbal task, which has been reproduced in at least four, independent studies (Logan and Fischman 2011, 2015; Schütz and Schack 2020; Weigelt et al. 2009), the survival of recency in a spatial WM task to date has only been shown in a single experiment (Schütz and Schack 2020). In the current study, we would, therefore, like to reproduce the effect to rule out the possibility that it was an incidental finding. The primary objective of the current study, however, is to test if the depletion of spatial WM resources scales with the amount of motor planning in a concurrent, sequential motor task. To this end, participants execute an ordered task with a lower and a randomised task with a higher percentage of motor planning in parallel to a spatial WM task.

## Results

**Fig. 1:**
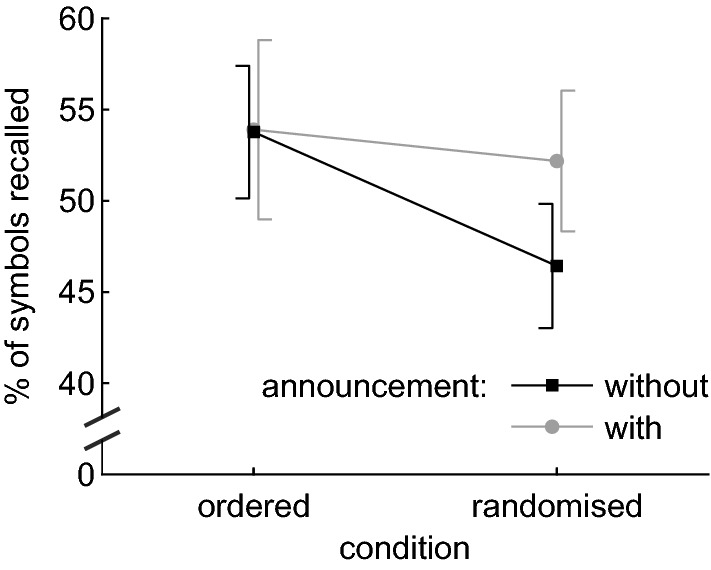
Recall performance in the ordered and randomised ‘condition’, split by ‘announcement’. Each data point represents the average across the factors ‘serial position’ and ‘repetition’. Error bars indicate 95% confidence intervals corrected for between subject variance

To compare recall performance of the four tasks, a repeated measures ANOVA (rmANOVA) with the factors ‘condition’ (ordered/randomised) and ‘announcement’ (without/with) was calculated. The main effect of ‘condition’ was significant, *F* (1, 27) = 5.074, *p* = 0.033, *η*^2^ = 0.013. Recall performance was better in the ordered (53.8%) than in the randomised (49.3%) tasks (see Fig. 1). The main effect of ‘announcement’ was not significant, *F* (1, 27) = 1.228, *p* = 0.278, *η*^2^ = 0.005. Recall performance did not depend on whether each upcoming drawer number was announced or participants had to memorise their position within the sequence. Importantly, the interaction of ‘condition’ × ‘announcement’ was not significant, *F* (1, 27) = 1.953, *p* = 0.174, *η*^2^ = 0.005, confirming that the main effect of ‘condition’ can be interpreted.

Since the graph strongly suggested an interaction, we calculated the Bayesian information criterion (BIC; cf. Raftery 1995) as a goodness of fit measure from the residual sum of squares, both for a 2-parameter model (main effects only) and a 3-parameter model (main effects + interaction). Lower BIC values indicate a better fit. There was no evidence in favour of a 3-parameter model (BIC = 55.71), but positive evidence (∆BIC = 4.17) in favour of a 2-parameter model (BIC = 51.54).

**Fig. 2:**
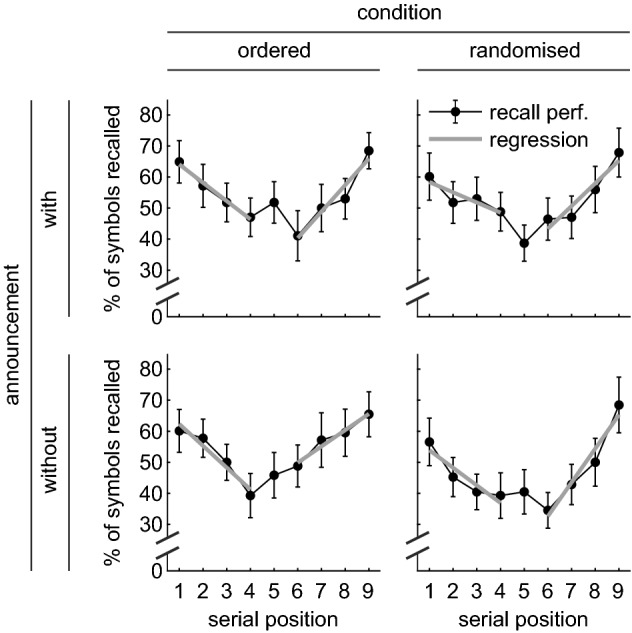
Recall performance plotted against ‘serial position’, split by the factors ‘condition’ (columns) and ‘announcement’ (rows). Each data point represents the average across ‘repetition’. Error bars indicate 95% confidence intervals corrected for between subject variance. Grey lines represent the linear model fitted to the first (primacy) and last (recency) four items

To test for *primacy* and *recency* effects, a linear model was calculated on the first and last four items of all four tasks, with dummy variables for ‘primacy/recency’, ‘condition’, ‘announcement’, and ‘serial position’. The main effects of ‘primacy/recency’, *t* (880) = 2.778, *p* = 0.006, *η*^2^ = 0.001, and ‘serial position’, *t* (880) = 3.060, *p* = 0.002, *η*^2^ = 0.003, were significant. Due to a significant cross-over interaction of ‘primacy/recency’ × ‘serial position’, *t* (880) = 3.777, *p* < 0.001, *η*^2^ = 0.071, however, these main effects could not be interpreted. The cross-over interaction indicates a significant primacy effect with a negative slope of − 5.5% per item and, more importantly, a significant recency effect with a positive slope of + 8.0% per item (see Fig. 2). Absolute value of both slopes did not differ, *t* (880) = 0.550, *p* = 0.582, *η*^2^ = 0.003. Neither of the interactions with ‘serial position’ was significant, which indicates that the slopes of primacy and recency were unaffected by ‘condition’ or ‘announcement’ and, thus, similar in all four tasks.

Polynomial contrasts were calculated on the ‘serial position’ curve of all tasks to evaluate the general pattern of results. The linear contrast was not significant, *F* (1,27) = 1.366, *p* = 0.253, *η*^2^ = 0.003; the quadratic contrast was highly significant, *F* (1,27) = 114.315, *p* < 0.001, *η*^2^ = 0.073. None of the interactions of the linear/quadratic contrast with ‘condition’ or ‘announcement’ was significant. The quadratic contrast indicates that recall performance of the first and last items was higher than for the intermediate items, suggesting a primacy and a recency effect. The absence of the interactions indicates that the pattern of results is similar in all four tasks.

Since participants had direct control over their movement time (MT) and the encoding time (ET) in the memory task (by opening and closing the drawers), the difference in recall performance for ‘condition’ might not reflect a different depletion of WM resources by the different motor tasks. Instead, it might result from a speed-accuracy trade-off (i.e. differences in MT) or different time spans participants invested in memorisation (i.e. differences in ET). Therefore, we tested for differences in MT and ET as a function of ‘condition’ and ‘announcement’.

A rmANOVA with the factors ‘condition’ and ‘announcement’ was calculated on the average MT (for opening and closing a single drawer). Only the main effect of ‘announcement’ was significant, *F* (1, 27) = 27.522, *p* < 0.001, *η*^2^ = 0.043. MT was shorter with (3926 ms) than without (4213 ms) an announcement of the next drawer number. The main effect of ‘condition’, *F* (1, 27) = 1.134, *p* = 0.296, *η*^2^ = 0.001, and the interaction of ‘condition’ × ‘announcement’, *F* (1,27) < 1, *p* = 0.978, *η*^2^ < 0.001, were not significant. MT did not differ between the ordered and the randomised condition.

A second rmANOVA was calculated on the average ET (for a single symbol). Again, only the main effect of ‘announcement’ was significant, *F* (1, 27) = 4.401, *p* = 0.045, *η*^2^ = 0.002. ET was shorter with (2520 ms) than without (2729 ms) an announcement of the next drawer number. The main effect of ‘condition’, *F* (1, 27) = 1.860, *p* = 0.184, *η*^2^ = 0.001, and the interaction of ‘condition’ × ‘announcement’, *F* (1, 27) = 1.982, *p* = 0.171, *η*^2^ = 0.002, were not significant. ET did not differ between the ordered and the randomised condition.

Even though differences in MT and ET were only found for ‘announcement’, but not for ‘condition’, we tested if MT and ET had any effect on recall performance. To this end, we calculated a generalised linear mixed-effects model (GLMM; binomial model, logit link) on the number of correctly recalled symbols in each of the four tasks, with ‘MT’, ‘ET’, ‘condition’, and ‘announcement’ as fixed effects, and ‘participant ID’ as a random effect. Neither the effect of ‘MT’, *z* = + 0.697, *p* = 0.486, *R*^2^_β*_ = 0.007, nor of ‘ET’, *z* = − 0.836, *p* = 0.403, *R*^2^_β*_ = 0.069, was significant. Recall performance was unaffected by MT and ET. The difference in recall performance does not result from a speed-accuracy trade-off or different time spans invested in memorisation.

The effect of ‘condition’ was highly significant, *z* = − 4.311, *p* < 0.001, *R*^2^_β*_ = 0.020. The effect of ‘announcement’ was not significant, *z* = + 0.308, *p* = 0.758, *R*^2^_β*_ < 0.001, but the interaction of ‘condition’ × ‘announcement’ was, *z* =  + 2.176, *p* = 0.030, *R*^2^_β*_ = 0.007. Since the interaction was hybrid, the main effect of ‘condition’ could be interpreted: Recall performance was better in the ordered (53.7%) than in the randomised (49.0%) tasks. Thus, the extended model replicated the result from the rmANOVA. To solve the interaction, individual GLMMs were calculated for the ordered and the randomised tasks. In the ordered tasks, the effect of 'announcement' was not significant, *z* = + 0.675, *p* = 0.500, *R*^2^_β*_ = 0.002, in the randomised tasks, it was, *z* = + 3.380, *p* < 0.001, *R*^2^_β*_ = 0.040. Recall performance was better with (53.4%) than without (46.5%) announcement of the next drawer.

To compare the percentage of reuse (PoR) between the four tasks, a model fitting approach was used. The BIC was calculated for three different models, to test whether a 5-parameter model was indicated for the description of the grasp angle data: a 2-parameter (linear) model, a 4-parameter (sigmoid) model and a 5-parameter (sigmoid + PoR) model.

A 4-parameter model (BIC = 266.33) fit the data significantly better than a 2-parameter model (BIC = 306.98), *t* (27) = 7.610, *p* < 0.001. As the BIC strongly penalises models with more parameters (i.e. 4-parameter model), a ∆ value of 40.64 provides very strong evidence in support of the 4-parameter model (Raftery 1995). A sigmoid function describes the grasp angle data better than a linear function.

A 5-parameter model (BIC = 262.06) fit the data significantly better than a 4-parameter model (BIC = 266.33), *t* (27) = 2.820, *p* = 0.009. A ∆ value of 4.28 provides positive evidence in favour of the 5-parameter model. A model incorporating a PoR parameter describes the grasp angle data better than a model which is restricted to a sigmoid grasp angle function.

**Fig. 3:**
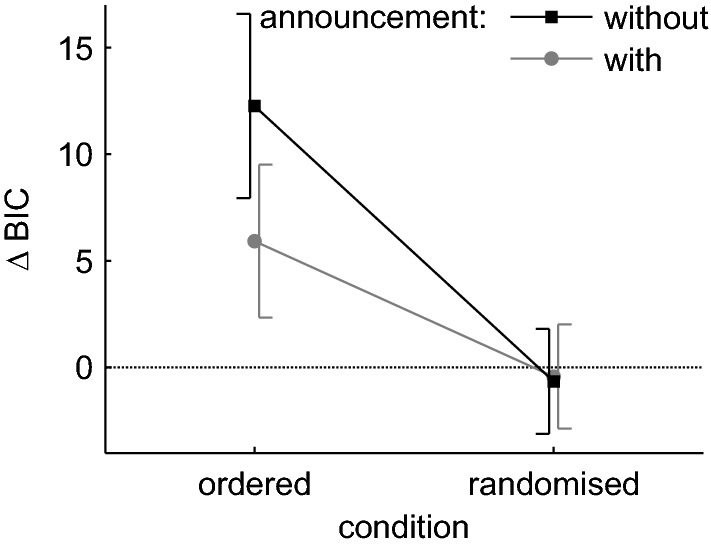
BIC ∆ values between the 4- and 5-parameter model, split by the factors ‘condition’ and ‘announcement’. Each data point represents the average across the factors ‘serial position’ and ‘repetition’. Error bars indicate 95% confidence intervals corrected for between subject variance

As Δ values between the 4- and 5-parameter model were available for each task, a rmANOVA with the factors ‘condition’ (ordered/randomised) and ‘announcement’ (without/with) was calculated. Only the main effect of ‘condition’ was significant, *F* (1, 27) = 18.592, *p* < 0.001, *η*^2^ = 0.145. There was strong evidence in favour of the 5-parameter model in the ordered tasks (Δ BIC = 9.09) but no evidence in the randomised (Δ BIC = − 0.54) tasks (see Fig. 3).

**Fig. 4:**
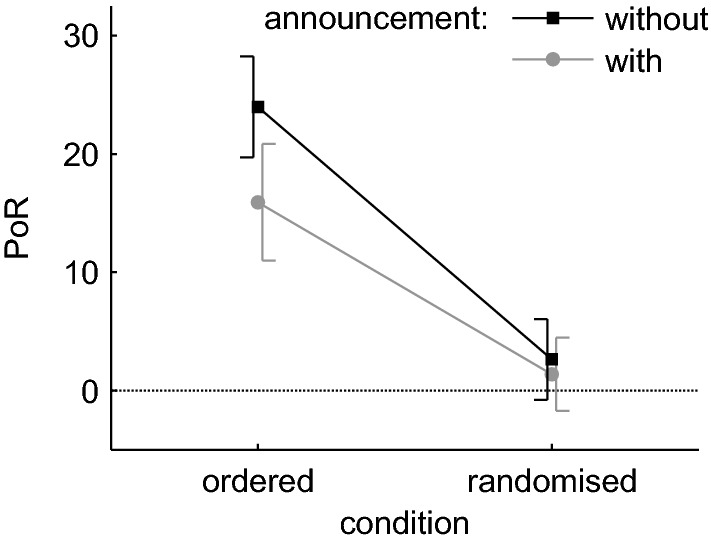
Percentage of (motor plan) reuse in the ordered and randomised ‘condition’, split by ‘announcement’. Each data point represents the average across the factors ‘serial position’ and ‘repetition’. Error bars indicate 95% confidence intervals corrected for between subject variance

To compare the PoR (as determined by the 5-parameter model) between tasks, a rmANOVA with the factors ‘condition’ (ordered/randomised) and ‘announcement’ (without/with) was calculated. The main effect of ‘condition’ was significant, *F* (1, 27) = 35.606, *p* < 0.001, *η*^2^ = 0.332. The PoR was higher in the ordered (20.0%) than in the randomised (2.0%) tasks (see Fig. 4). The main effect of ‘announcement’ was also significant, *F* (1, 27) = 6.401, *p* = 0.018, *η*^2^ = 0.022. The PoR was higher (13.3%) if participants executed the sequences on their own and lower (8.7%) if each upcoming drawer number was announced by the experimenter (see Fig. 4).

The interaction of ‘condition’ × ‘announcement’ was only close to significance, *F* (1, 27) = 4.199, *p* = 0.050, *η*^2^ = 0.012, and ordinal, so both main effects could be interpreted independent of the interaction. If the interaction were significant this would indicate that (1) the effect of ‘announcement’ depended on ‘condition’ and that (2) the announcement had a larger effect on the PoR in the ordered (8.1%) than in the randomised (1.3%) condition.

## Discussion

In the current study, we asked if the available resources in working memory (WM) depended on the percentage of (motor plan) reuse (PoR) in a concurrent motor task. To this end, participants executed two sequential motor tasks: a randomised task with a presumably lower and an ordered task with a presumably higher PoR. To verify the expected PoRs in both tasks, hand pro/supination was measured as a dependent variable. In parallel to the motor task, participants conducted a spatial WM task (memorise symbols in a 4 × 4 matrix). Recall was measured as a second dependent variable. We expected a systematic interference of motor planning on spatial recall, that is, a worse recall performance in the randomised task, which requires more motor planning.

Based on the hand pro/supination values, we applied a 5-parameter model to estimate the PoR in the two different motor tasks. The repeated measures ANOVA (rmANOVA) calculated on the PoR values showed a significant main effect of ‘condition’: the PoR in the ordered task was significantly higher (20.0%) than in the randomised task (2.0%), indicating that the experimental manipulation was working. Despite this significant difference, one should note that, even in the ordered sequential task, a major fraction (80.0%) of each motor plan was created by re-planning. A similar result (84.4% re-planning) has been reported for an ordered sequential task previously (Schütz and Schack 2019).

The *plan-modification hypothesis* (Schütz and Schack 2013) states that the PoR in a sequential task is closely linked to (1) the relative cognitive cost of motor planning and (2) the relative mechanical cost of motor execution. If the relative cost of motor planning and execution were approximately equal, a PoR of 50% would be expected. If one cost factor was more relevant than the other, the PoR would shift to one side (cf. Schütz and Schack 2019, Fig. 1b). The PoR of 80% found in the current study suggests that the mechanical cost of motor execution exceeds the cognitive cost of motor planning. To get a real estimate of the relative costs, however, one would need to conduct a series of experiments with systematic changes in mechanical cost, while measuring the PoR.

As the main result of the current study, the rmANOVA calculated on the average recall performance showed a significant main effect of ‘condition’: recall was better in the ordered (53.8%) than in the randomised (49.3%) tasks. Tasks with a higher percentage of motor planning have a larger disruptive effect on spatial WM than tasks with a lower percentage, indicating that the (spatial) WM resources depleted by the concurrent motor task scale with the amount of motor planning. A nice feature of the current study was that only the amount of motor planning varied, while overall motor execution was the same in both tasks.

A qualitatively similar result was reproduced with the generalised linear mixed-effects model (GLMM), which included fixed effects for movement time (MT) and encoding time (ET). Neither the effect of MT or ET were significant, which indicates that the difference in recall performance does not result from a speed-accuracy trade-off or different time spans participants invested in memorisation. Unlike the rmANOVA, the GLMM showed a significant interaction of ‘condition’ × ‘announcement’. This interaction resulted from a worse recall performance in the randomised task without (46.5%) than in the randomised task with (53.4%) announcement. Presumably, this decline in recall performance reflected the additional WM load for the retention of the randomised sequence.

One can speculate which memory process was disrupted by the motor task: encoding, maintenance, or retrieval. Since retrieval happened well after the end of the motor task, was self-paced, and identical in all four tasks, it is the least likely candidate. There was some overlap between encoding and movement execution (specifically, the end of drawer opening and the beginning of drawer closing), but encoding, too, was self-paced (participants had direct control over ET) and recall performance in the GLMM was unaffected by ET. Thus, we consider maintenance the most likely candidate: After the first drawer, movement planning took place while spatial WM information had to be retained. Several studies have demonstrated that the maintenance of visual WM content is affected by the execution of reaching movements executed well after encoding (Hanning and Deubel 2018; cf. Heuer et al. 2020; Heuer and Schubö 2018).

An isolated effect of motor planning on spatial WM, as shown in the current study, has previously been measured by Spiegel et al. (2013). The authors asked participants to (1) reach for an object, (2) plan a placing movement to the left/right side on a visual cue, (3) memorise a spatial matrix, and (4a) execute the pre-planned movement or (4b) re-plan to execute a movement in the opposite direction (signalled by an auditory cue). Therefore, motor execution was comparable in both conditions, while the percentage of motor planning was either 0% or 100% (Quinn and Sherwood 1983). In the re-planning condition, spatial WM performance degraded from 3.07 items to 2.85 items (Δ = − 0.22 items).

In the current study, we showed that not only a binary switch from 0 to 100% motor planning has a disruptive effect on spatial WM, but also a fractional increase in re-planning. The increase from 80 to 98% re-planning degraded WM performance from 3.23 items to 2.96 items and, thus, had a similar disruptive effect (Δ = − 0.27 items) on spatial WM as the full re-planning in the study by Spiegel et al. (2013). We can rule out the hypothesis that the disruptive effect reflects a general cost for the reconfiguration of a motor plan (independent of the PoR), since there should be no effect of ‘condition’ in the current study if this was the case. A potential reason for the comparable effect sizes could be the higher complexity of the movement in the current study (reach, open, close, return vs. reach only).

Both studies also differed in the memory task: symbols were presented simultaneously by Spiegel et al. (2013), but sequentially in the current study. In the *multicomponent model* (Baddeley and Hitch 1974), simultaneously presented items are stored exclusively in the *episodic buffer* (Baddeley 2000). In contrast, sequentially presented items are partly stored in the *visuospatial sketchpad* (Baddeley 2001). The comparatively large disruptive effect of partial re-planning on WM in the current study could be explained if motor planning shared more common resources with the visuospatial sketchpad than with the episodic buffer. Indeed, a number of studies indicate a close link of movement execution to spatial WM (Lawrence et al. 2001, 2004; Logie and Pearson1997).

More recent findings, however, also indicate a link to the episodic buffer: if a verbal recall task and a motor task are combined, the *recency effect* (attributed to the *episodic buffer;* Baddeley 2000) is lost (Logan and Fischman 2011, 2015; Weigelt et al. 2009). If either a verbal or a spatial recall task were combined with a motor task, recency was absent in the verbal, but present in the spatial task (Schütz and Schack 2020). This differential interference has important implications for current models of WM, which is why, in the current study, we asked if the spatial recency effect, to date only found in a single study, could be reproduced. Both the contrast and regression analyses on the recall data showed a clear recency effect in all four experimental conditions.

This finding is in stark contrast to the predictions of the multicomponent model, which claims the episodic buffer as a limited-capacity store of the central executive that, due to its *non-domain-specific encoding*, can integrate information from the two domain-specific sub-systems (Baddeley 2003). Therefore, the model cannot account for a differential interference of the same motor task on verbal and spatial recency. A similar problem arises for the unitary, *object-oriented episodic record model* (Jones et al. 1995, 2004; Macken et al. 2015), which assumes a common representation of verbal and spatial information. The *changing state hypothesis* (Jones et al. 1992), which is part of the model, claims that interference is determined by the degree to which two tasks contain serial order cues. Since order cues in the motor task were identical in the study by Schütz and Schack (2020), the model as well fails to account for the differential effects.

A new study (Joseph and Morey 2021) found neither convincing evidence for a multicomponent nor for a unitary WM model in a complex span task and, thus, better matches our current findings. The authors hypothesised that interference primarily reflects a reformatting of sensory representations into motor representations (Joseph and Morey 2021; Myers et al. 2017). Differential effects result from specialised sensory and motor systems instead of specialised WM stores. Thus, interference in our study would be the result of a shift of attention between the different fractions of reconfiguration of the reaching motor plan and the automatic reconfiguration of the WM representations into motor output. Since visual information is less directly convertible to a motor output than verbal information (which is actively reformatted to speech; Joseph and Morey 2021), interference of motor re-planning with spatial recency could be reduced.

As a last minor research question, we asked whether the announcement of drawer numbers would affect recall performance and motor planning. A negative effect of ‘announcement’ on recall performance could indicate a deduction of attentional resources (Cowan 2001; Kahneman 1973) or the creation of a serial object record that interferes with WM (Jones et al. 1992; Jones 1993; Macken et al. 2015), a positive effect could indicate a facilitation of memory retention. The main effect of 'announcement' on recall performance was not significant, supporting neither interpretation. The interaction of ‘condition’ × ‘announcement’ was significant in the GLMM only, presumably reflecting the additional cognitive load for the maintenance of the randomised sequence.

With respect to PoR, a positive effect of ‘announcement’ could indicate a deduction of attentional resources from planning or a facilitation of plan retention, a negative effect a decay of the former plan due to the added delay between trials (Jax and Rosenbaum 2009). Results showed a negative effect of ‘announcement’ on the PoR, which favours the decay hypothesis. However, time courses do not fit the decay hypothesis: in a drawer study (Schütz and Schack 2013) similar to the current, time for announcing drawer numbers was measured as ~ 680 ms. While path plans indeed decay within 1000 ms (Jax and Rosenbaum 2009), posture plans are more stable: Weigelt et al. (2009) had participants open a drawer, extract a cup, memorise a letter, return the cup, and step back. These steps should take at least 8 s, yet participants exhibited a significant hysteresis effect. Therefore, one would not expect the minor delay for the announcement to cause the significant decrease in PoR found in the current study.

As an alternative explanation for the negative effect of ‘announcement’ on the PoR, one could speculate that, due to the announcement, participants no longer perceived the task as a closed sequence but as divided into individual drawers (despite the strictly consecutive drawer numbers in the ordered tasks). Indeed, when Schütz and Schack (2019) asked participants to open either every drawer or every second drawer in an ordered sequence, PoR in the second task was significantly reduced, even though the delay between two drawers was the same. This result indicates that the PoR does not only depend on cognitive and mechanical cost (Schütz and Schack 2013), but also on the perceived cohesion between subsequent movements, which might be affected by context factors (e.g. digits, distance, or individual announcement). The negative effect of ‘announcement’ on the PoR might, therefore, reflect such a loss of perceived cohesion.

In the current study, we asked whether a sequential task with less motor re-planning would deplete fewer WM resources. To this end, we asked participants to execute a spatial WM task in parallel to either (1) a randomised task with a high amount of motor re-planning or (2) an ordered task with a lower amount of motor re-planning. Recall performance in the memory task was measured as a dependent variable; hand posture was measured to validate the experimental approach. Results showed less motor re-planning and better recall performance in the ordered task, confirming our hypothesis. As a second, minor result, we reproduced a clear recency effect in all four experimental conditions in our spatial WM task. As several previous studies found a loss of recency when combining a verbal WM task with a motor task, this finding suggests a differential effect of a concurrent motor task on verbal vs. spatial WM, which is not accounted for by several current WM models.

## Methods

### Participants

Twenty-eight students (12 male, age 23.8 ± 3.5 years) from Bielefeld University participated in the experiment. All participants were right handed (handedness score 0.99 ± 0.05) according to the revised Edinburgh inventory (Oldfield 1971). Participants reported no known neuromuscular disorders and were naive to the purpose of the study. Each participant read a detailed set of instructions on the task and gave written informed consent before the experiment. The study was conducted in accordance with the 1964 Declaration of Helsinki [latest revision in Fortaleza (World Medical Association 2013)] and was approved by the local ethics committee.

### Apparatus

**Fig. 5:**
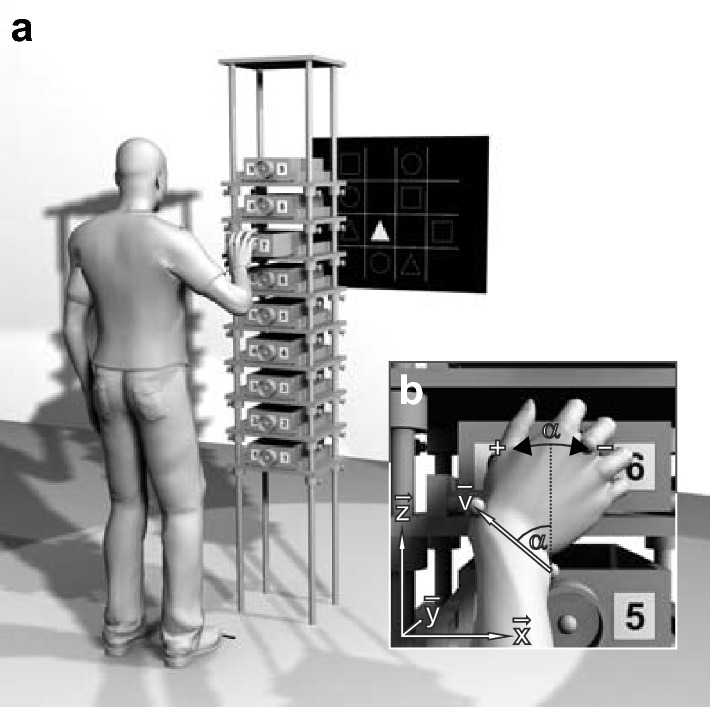
**a** Schematic of the experimental setup. Drawer height, drawer spacing, and participant’s positions are scaled based on shoulder height and arm length. Two strips of black tape mark the participant's positions in front of the setup. **b** Pro/supination angle α at the moment of drawer grasp. The projection of the wrist vector **v** onto the drawer face (**x**–**z**-plane) is used to calculate α

The apparatus used was a tall metal frame (222 cm high, 40 cm wide, 30 cm deep) with nine wooden shelves (see Fig. 5a). A wooden drawer (8.5 cm high, 20 cm wide, 30 cm deep; pullout range 21.5 cm) was placed on each shelf. At the centre of each drawer front, a grey plastic ring (7 cm diameter, 4 cm deep) was affixed. On both sides of this handle, the drawer number was depicted, ranging from 1 (lowest) to 9 (highest).

Stimuli were displayed by a Canon LV-X6 projector (Canon Inc, Tokyo, Japan). The projection screen (92 cm high, 115 cm wide) was located 255 cm behind the drawer faces and 45 cm to the left of the drawer centre (see Fig. 5a). The upper edge of the screen was 200 cm above floor level. Stimulus presentation was controlled by Presentation^®^ 18.1 (Neurobehavioral Systems, Inc., Albany, CA). A stimulus was presented every time a drawer was opened (pullout > 14 cm) and switched off once the drawer was closed (pullout < 7 cm). As stimuli, 4 × 4 symbol matrices (80 cm high, 80 cm wide) were used (see Fig. 5a). Three different types of symbols (12 cm high, 12 cm wide) were presented: circles, triangles, and squares.

### Preparation

Retro reflective markers were attached to ten bony landmarks on the right arm and thorax of the participants (for all positions, see Schütz and Schack 2013, Table 1). Four markers were used for posture calculations: most cranial point of the acromion, *Articulatio acromioclaviculare* (AC), *radial* (RS) and *ulnar* (US) *styloid process*, and top of the third metacarpal, *Os metacarpale tertium* (MC). The approximate height of the shoulder joint centre (0.97 × *height of AC*) and the length of the arm (||AC − RS||) were measured in a T-pose (arms extended sideways, palms pointed forward).

The centre of drawer #7 was aligned with the height of the shoulder joint centre. Drawer spacing was set to 0.25 × *arm length*. Participants’ position was standardised with the shoulder joint centre 1.00 × *arm length* in front of the drawer face and 0.33 × *arm length* to the left of the drawer centre (see Fig. 5a). This reference position was marked by two strips of adhesive tape (point of the toes and median plane of the body).

### Motor task

The experiment was split into four tasks, presented in balanced order to minimise order effects in the data (i.e. fatigue, familiarisation, or learning). A single task consisted of six sequences of nine trials (plus two warm-up sequences). A single trial was defined as the opening and closing of one drawer. Each trial started from an initial position, with the palm of the hand touching the thigh. Participants had to (1) raise the arm to the drawer, (2) grasp the handle with a five-finger grip (see Fig. 5b), (3) open the drawer, (4) memorise a depicted symbol and its position, (5) close the drawer, and (6) return to the initial position.

In Task 1, participants performed six ordered sequences of the nine drawers, three in ascending and three in descending direction (2 directions × 3 repetitions × 9 drawers: 54 trials). The sequence of the directions was randomised. The experimenter announced each upcoming drawer number as soon as the arm was back in the initial position. Participants did not have to memorise their position within the sequence.

In Task 2, participants also performed six ordered sequences of the nine drawers (2 directions × 3 repetitions × 9 drawers: 54 trials). However, the experimenter did not announce individual drawer numbers but only the direction of the upcoming sequence (‘top to bottom’/‘bottom to top’). Participants had to execute the nine trials of each sequence on their own and memorise their position within the sequence.

In Task 3, participants performed six randomised sequences of the nine drawers (6 sequences × 1 repetition × 9 drawers; 54 trials). A list of pseudo-random (Mersenne twister algorithm, Matsumoto and Nishimura 1998) permutations was created before the experiment. From the list, the experimenter announced each upcoming drawer number as soon as the arm was back in the initial position. Participants did not have to memorise their position.

In Task 4, participants first memorised a single randomised sequence of the nine drawers by heart. To this end, the warm-up was preceded by a practise phase, in which participants first memorised the numerical sequence and then executed a minimum of eight practise sequences on the setup (without a concurrent memory task). Only when participants could reliably reproduce the randomised sequence, the real task started. Participants performed six sequences of the nine drawers (1 sequence × 6 repetitions × 9 drawers; 54 trials) on their own and had to memorise their position within the sequence. Sequences were created before the experiment (identical to Task 3) and altered between participants.

At the beginning of each task, participants’ position in front of the apparatus was standardised based on the reference position. Each task started with two warm-up sequences. Participants had a resting period of 30 s between sequences and of 2 min between tasks. The entire experiment lasted 120 min.

### Memory task

For the memory task, sequences of nine symbols (three different types: circle, triangle, and square) were presented in a 4 × 4 spatial matrix. The frequency of symbol type and position was controlled for within participants. The frequency of each symbol type at each of the 16 positions was controlled for across participants. Direct repetitions of the same symbol in a sequence were allowed, but not twice in a row. In each sequence, any matrix position could occur only once. That way, participants could sketch all recalled symbols into a single matrix after each sequence.

The memory task was conducted in parallel to the four different motor tasks. While the motor tasks differed, the memory task was always the same: Each time a drawer was opened one of the three different symbols appeared in one of the 16 different matrix positions. Participants had direct control over the onset/offset of the memory stimulus by opening/closing of the drawer. At the end of each sequence, participants had to sketch the recalled symbols (up to nine) at their correct positions into an empty matrix template. Symbols could be reported in any order (free recall).

### Kinematic analysis

Movement data were recorded by a Vicon MX (Vicon Motion Systems, Oxford, UK) motion capture system. Marker trajectories were reconstructed in Vicon Nexus 2.6.1, labelled manually, and exported to MATLAB (2015a, The MathWorks, Natick, MA) for data analysis. The laboratory’s coordinate system was defined with the **x**-axis pointing to the right, the **y**-axis pointing to the front and the **z**-axis pointing upwards while standing in front of the apparatus (see Fig. 5b).

To identify the moment of drawer grasp for each trial, the **y**-component (perpendicular to the drawer face, see Fig. 5b) of the *capitulum* marker (MC) was analysed. Its trajectory started from a low initial value (the initial position) and exhibited two local maxima before returning to the initial value. The first local maximum, which corresponded to the moment of drawer grasp, was used to calculate the pro/supination angle α.

For the calculation of α, the wrist axis was projected onto the drawer face (**x**–**z**-plane, see Fig. 5b). A direction vector **v** was defined, pointing from US to RS: **v** = RS–US. From the vector components v_x_ and v_z_, the pro/supination angle α was calculated with the four-quadrant inverse tangent function of MATLAB. It was zero when the back of the hand pointed directly to the right (**v** pointed directly upward). Pronation of the hand caused an increase, supination a decrease of the pro/supination angle.

Encoding time (ET) was calculated based on the **y**-component of MC. Presentation time of the stimuli depended directly on the opening and closing of the drawers: Stimuli were switched on at a pullout value of 140 mm and switched off at a pullout value of 70 mm. While a drawer was grasped, MC moved in parallel with the drawer and, thus, could be used as a proxy for drawer pullout. For each individual drawer opening, we calculated the difference between the (constant) **y**-value of MC measured at the moment of drawer grasp, and its (variable) **y**-trajectory (MC_y,diff_ = MC_y,grasp_ – MC_y,traj._). Stimulus onset was defined as the moment when MC_y,diff_ exceeded 140 mm while opening the drawer, stimulus offset as the moment when MC_y,diff_ fell below 70 mm while closing the drawer.

Movement time (MT) was calculated based on the absolute velocity of MC. Individual components of MC were first smoothed two times by a moving average (with a time window of 100 ms) and then differentiated. Absolute velocity was calculated from the individual components. For each of the four phases of a drawer opening (reach, open, close, return) the maximum velocity was measured. Onset/offset times of each movement phase were defined as the moments when absolute velocity fell below 5% of the maximum velocity. Durations of the four movement phases were then added to calculate the total movement duration at each drawer.

### Percentage of reuse

In theory, to determine the size of the motor hysteresis effect in the sequential tasks (Tasks 1 and 2), a repeated measures ANOVA (rmANOVA) on the pro/supination angle α could be calculated. The size of the main effect of the factor ‘direction’ would indicate the size of the hysteresis effect.

This approach, however, is not viable for the randomised tasks (Tasks 3 and 4). In Task 3, six pseudo-random sequences of trials were tested for each participant. Therefore, the probability for the central drawers to be grasped (at least) once in a descending and once in an ascending sequence was high. Even for the central three drawers (4, 5, and 6), however, complete data sets were only available for 23 of the 28 participants. In Task 4, a single pseudo-random sequence was tested six times for each participant. Therefore, drawers were grasped either in an ascending or in a descending sequence each time, rendering the analysis impossible.

To measure the percentage of reuse (PoR) in all four tasks, we used a model fitting approach (Schütz and Schack 2019). A five-parameter model was used to capture the pattern of results found for the pro/supination angle α. Four parameters were required to model the optimal pro/supination angle α at each drawer as a sigmoid (*tanh*) function: its range (from lowest drawer to highest), its steepest slope, and the x- and y-offset of its origin.

The fifth parameter was the PoR between subsequent drawers. A PoR of 100% would result in a direct repetition of the previous grasp angle, a PoR of 0% would result in the optimal pro/supination angle α for each drawer. A PoR of 20% creates a weighted average of the previous grasp (20%) and the optimal grasp (80%). The modelling algorithm was provided with the 24 sequences of drawer numbers of each participant. That way, a persistence to the previous grasp type could be modelled not only in the ordered sequences of Tasks 1 and 2, but also in the randomised sequences of Tasks 3 and 4.

Model parameters were fitted to the measured data of individual participants using a least squares optimisation algorithm (Levenberg 1944; Marquardt 1963). Four parameters were used to model the optimal pro/supination angles and were identical for all four tasks. The fifth parameter, the PoR, was calculated individually for each task. Calculated PoR values were then used as the dependent variables for the statistical analyses.

To evaluate whether a five-parameter model was indicated for the modelling of the grasp angle data, we calculated the Bayesian information criterion (BIC, cf. Raftery 1995) as a goodness of fit measure for a 2-parameter (linear) model, a 4-parameter (sigmoid optimal grasp) model, and a 5-parameter (sigmoid optimal grasp plus an individual PoR for each task) model. The BIC was calculated individually for each task.

### Statistical analyses

rmANOVAs and polynomial contrast analyses were calculated in SPSS (28, IBM Corp., Armonk, NY) to apply the Greenhouse–Geisser correction. Linear models and generalised linear mixed-effects models (GLMMs) were calculated in R (4.1.1; R Core Team, 2021). GLMMs were calculated with the lme4 package (Bates et al. 2015). Effect sizes for the GLMMs were calculated with the r2glmm package (Jaeger et al. 2017), using the method of Nakagawa and Schielzeth (2013).

For the memory task, the percentage of symbols recalled correctly (i.e. symbol and position correct) was defined as the dependent variable. To compare recall performance between the four tasks, a rmANOVA was calculated. Experimental ‘condition’ (ordered/randomised sequences) and drawer number ‘announcement’ (without/with) were used as within subject factors. The within subject factors ‘serial position’ (of the nine consecutive symbols in one sequence) and ‘repetition’ (of the six sequences in each task) were averaged to reduce variance. Additional rmANOVAs with the same design were calculated to compare MT and ET between the four tasks. To this end, the average time for the opening and closing of a (single) drawer and the average presentation time of a (single) symbol on screen were used as dependent variables.

To test if differences in recall performance resulted from a speed-accuracy trade-off (i.e. differences in MT) or different time spans participants invested in memorisation (i.e. differences in ET) a GLMM (binomial model, logit link) was calculated on the number of correctly recalled symbols in each of the four tasks, with 'MT', ‘ET’, ‘condition’, and ‘announcement’ as fixed effects, and ‘participant ID’ as a random effect.

To evaluate the effect of the concurrent motor task on the primacy and recency portion of the serial position curve, the recall percentage was split by the within subject factor ‘serial position’ for each task. Then, a linear model was calculated on the first (primacy) and last (recency) four items of all four tasks (Schütz and Schack 2020; Weigelt et al. 2009), with dummy variables for ‘primacy/recency’, ‘condition’, ‘announcement’, and ‘serial position’. A negative slope for the first four items would indicate a primacy effect, and a positive slope for the last four items a recency effect.

We further calculated linear and quadratic contrasts to evaluate the general pattern of results in the serial position curves (Farrand et al. 2001; Jones et al. 1995). If only a primacy but no recency effect were present, we expected a significant linear contrast (decrease in memory performance throughout the sequence). If both a primacy and a recency effect were present, we expected a significant quadratic contrast (high performance in the initial and final portion of the serial position curve, lower performance in between).

For the motor task, the PoR (from the model) was defined as the dependent variable. To compare the PoR between the four tasks, a rmANOVA was calculated. The experimental ‘condition’ (ordered/randomised sequences) and drawer number ‘announcement’ (without/with) were used as within subject factors.

## Data Availability

The datasets generated and analysed during the current study are available from the corresponding author on reasonable request.
